# Evaluation of iron and manganese-coated pumice application for the removal of as(v) from aqueous solutions

**DOI:** 10.1186/1735-2746-9-21

**Published:** 2012-12-10

**Authors:** Leila Babaie Far, Bubak Souri, Masoumeh Heidari, Roshan Khoshnavazi

**Affiliations:** 1 Department of Environmental Sciences, Faculty of Natural Resources, University of Kurdistan, Sanandaj, Iran; 2 Department of Environmental Sciences, Islamic Azad University, Sanandaj, Iran; 3 Department of Chemistry, Faculty of Basic Sciences, University of Kurdistan, Sanandaj, Iran

**Keywords:** Arsenate, Iron-coated pumice, Manganese-coated pumice, Kinetics

## Abstract

Arsenic contamination of water has been recognized as a serious environmental issue and there are reports on its epidemiological problems to human health. The objective of this study was to evaluate the performances of iron-coated pumice and manganese-coated pumice as the adsorbents for removing arsenate from aqueous solutions. The effect of various parameters such as adsorbent dose, contact time, pH and initial concentration on removal efficiency of arsenate were evaluated in batch mode. The data obtained from the kinetic studies were analyzed using kinetic models of pseudo-first-order and pseudo-second-order. In addition, two isotherm models of Freundlich and Langmuir were used to fit the experimental data. The results showed that the optimum dosage of iron-coated pumice and manganese-coated pumice for arsenate removal were 40 and 80 g/L whereas the adsorption process reached equilibrium after 80 and 100 min, respectively. The maximum removal efficiency of arsenate using the two adsorbents were both recorded in pH=3 as the removal efficiency gradually declined following every increase in pH values of the solution. Iron-coated pumice also showed to have high removal efficiency when the initial concentration of arsenate was high while the low concentration of arsenate was efficiently removed by manganese-coated pumice. Moreover, it was depicted that the adsorption kinetics by both adsorbents followed pseudo-second order equation and the uptake data of arsenate were well fitted with Langmuir isotherm model. Therefore, it could be concluded that iron and manganese-coated pumice could be considered as suitable adsorbents for arsenate removal from aqueous solutions.

## Introduction

Among inorganic contaminants, the metalloid arsenic has been widely studied due to its potential adverse to human health [1]. Arsenic in natural waters occur in both organic and inorganic forms while its inorganic forms are more toxic to human health and commonly occur as arsenate (As(V)) and arsenite (As(III)). pH and redox potential are the most important parameters in domination of As(V) and As(III) in environment (Pokhrel and Viraraghavan, [2]).

The presence of high levels of arsenic in natural water resources is considered as a global problem while countries of Bangladesh, India, USA, China, Chile, Taiwan, Mexico, Argentine, Poland, Canada, Hungary, New Zealand, Japan and Iran have reported its high amounts in water resources [3-5]. Because of the high toxicity and carcinogenic effect of arsenic to human, the World Health Organization (WHO) and the United States Environmental Protection Agency (USEPA) have recommended a Maximum Contaminant Level (MCL) of 10 μg/L for arsenic in drinking water [6].

Until now the numerous and effective technologies has been developed in order to remove arsenic from water. The major techniques for arsenic removal are: oxidation, coagulation, sorption, precipitation/coprecipitation, ion exchange and reverse osmosis [7] as adsorption methods are much important because of their relatively low cost and easy operation (Do, [4,8]).

The use of natural geomaterials as adsorbents like sand, olivine and quartz as support media which are amended with coating materials to enhance their adsorptive capacity for arsenic removal have been widely considered in recent years ( [9]; Kundu and Gupta, [10-12]). Therefore, in this study granular particles of pumice igneous stone were applied based on their capability to remove heavy metals [13-15] after being coated with iron and manganese as possible adsorbents for removal of As(V) from aqueous solutions. In fact, the main scope of this study was to examine various parameters such as adsorbent dosage, pH, initial concentration of As(V), contact time, sorption kinetics and equilibrium isotherm during removal procedure.

## Materials and methods

### Chemicals

All used chemicals in this study were reagent grade from Merck (Germany), while sodium arsenate (NaHAsO_4_.7H_2_O) was from Analar (England). As(V) stock solution was prepared by dissolving of sodium arsenate in double distilled water. pH of the solutions were adjusted to the desired values using either NaOH or HNO_3_ dilute solutions.

### Iron-coated pumice (ICP) and manganese-coated pumice (MCP)

Pumice stone was collected from a mine in Qorveh region of Kurdistan province in western Iran, where plenty of such mines are available. Prior to coating Fe and Mn on pumice surface, the stone was crushed and sieved through No. 40 and 50 mesh size sieves in order to produce particle size fractions of 0.3 and 0.42 mm. Then, the obtained particles were immersed in 37% HCl for 24hrs and washed several times using distilled water. In order to prepare ICP and MCP, solutions of 0.5 M Fe(NO_3_)_3_.9H_2_O and Mn(NO_3_)_2_ were adjusted to pH=12 and 8, respectively, by adding NaOH and mixed acid washed pumice particles. The beakers containing slurry were placed in a static state in laboratory temperature (25±1°C) for 72hrs and then dried in the oven at 110°C for 24hrs. Finally, the dried particles were washed three times by distilled water and then oven dried again at 110°C for 24hrs [16].

Surface mineralogy of ICP and MCP was determined by an X-ray diffractometer (XRD, model APD 2000, Ital Structures, Italy). A scanning electron microscopy (SEM, model JSM-840A, JEOL, Japan) was used for observation of the natural pumice and MCP surface morphologies. The specific surface area of the two adsorbents was measured using BET gas adsorption method in Research Institute of Petroleum Industry (RIPI) in Tehran.

### Batch experiments

As(V) removal reactions were performed in batch mode in 100 mL erlenmeyer flasks as sorption reactors. Parameters evaluated and their related ranges were adsorbent dosage (10–100 g/L) (pretest’s results showed that dosage lower than 10 g/L were unable to remove As(V) efficiency), contact time (5–360 min) (Tripathy and Raichur, [17]; Barakat and Sahiner, [18]), pH (3–11) (Tripathy and Raichur, [16,17]) and initial As(V) concentration (10–1000 μg/L) [16]. To get the reactions completed, all samples were placed in incubation shaker (model Certomat® BS-1, Sartorius, Germany) and mixed at 200 rpm under constant temperature (22±1°C). The samples were filtered through a 0.45 μm membrane filter and centrifuged at 4000 rpm. Then the filtrate was acidified with HNO_3_ and stored at 4°C until residual arsenic in the sample was measured by graphite furnace of an atomic absorption spectrophotometer (GFAAS, model Phoenix-986, Biotech, England).

The amount of arsenate adsorbed by to the adsorbents was calculated using equation (1) [19,20]:

(1) $$q_{e}=\frac{\left(C_{0}-C_{e}\right)V}{M}$$

Whereas q_e_ is the amount of adsorbed As(V) per unit mass of adsorbent (mg/g), C_0_ and C_e_ are initial and residual As(V) concentrations (mg/L), respectively, V is the volume of the solution (L) and M is adsorbent dose (g).

All experiments were conducted in duplicate and the mean values were reported.

### Sorption kinetics

The sorption kinetics of As(V) onto ICP and MCP were examined in various time intervals from 5 to 360 min and evaluated using Lagergren pseudo-first-order and Ho’s pseudo-second-order models [12]. The Lagergren pseudo-first-order model is expressed as equation (2):

(2) $$q_{t}=q_{e}\left(1-e^{-k_{1}t}\right)$$

The Ho’s pseudo-second-order rate equation is expressed as equation (3):

(3) $$q_{t}=\frac{q_{e}^{2}k_{2}t}{1+q_{e}k_{2}t}$$

Whereas q_t_ and q_e_ are the amount of arsenate adsorbed at any time t (min) and equilibrium time (mg/g), respectively. k_1_ and k_2_ are constant rates of the pseudo-first-order (1/min) and the pseudo-second-order adsorptions (g/mg.min).

Parameters of kinetic models were determined by trial and error non-linear method using MATLAB software [19]. Fitness of kinetic models to the experimental data was evaluated based on root mean square error (RMSE) values as the smaller RMSE value indicated the better curve fitting.

### Sorption isotherms

Sorption isotherm were experimented by varying amounts of adsorbents ranging from 1 to 80 g/L at a constant initial As(V) concentration of 1000 μg/L under pH=7 and 24hrs contact time. Freundlich and Langmuir models are the most commonly used two-parameter isotherms for single-solute adsorption [19,21]. The basic assumption of the Langmuir isotherm is that adsorption happens at specific homogeneous sites and forms a monolayer [21]. The Langmuir model is given by equation (4):

(4) $$q_{e}=\frac{q_{m}bC_{e}}{1+bC_{e}}$$

Whereas q_m_ represents the maximum amount of adsorbed arsenate per unit mass of sorbent (mg/g), b is the Langmuir constant (L/mg), related to the energy of adsorption and increases with the increase of adsorption bond strength.

The empirical Freundlich model is based on sorption at heterogeneous surface and is given by equation (5):

(5) qe=kfCe1n

Whereas k_f_ is the Freundlich constant (mg/g) (mg/L)^-1/n^, indicative of the relative adsorption capacity of adsorbent and the constant n is Freundlich equation exponent [19].

## Results

### Adsorbents characterization

**Figure 1:**
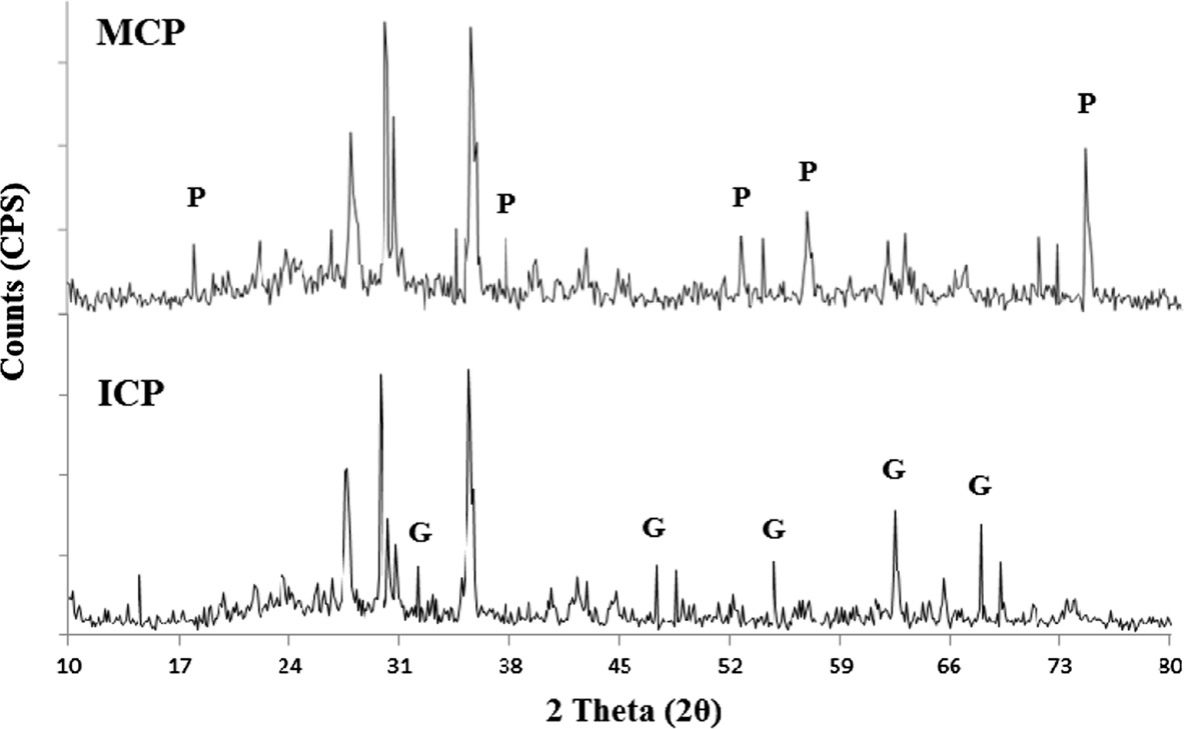
**X-ray diffraction spectrums of MCP and ICP.** (P: Pyrolusite, G: Goethite).

**Figure 2:**
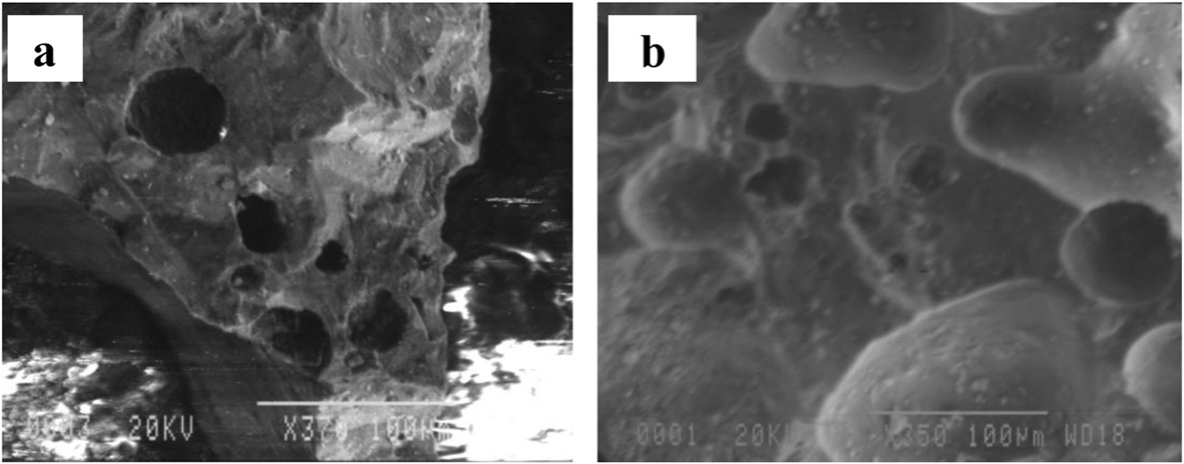
SEM images of natural pumice (a) and MCP (b).

The XRD spectrums obtained for MCP and ICP using Cu Kα radiation (λ= 1.5406 Å) at scan range of 10-80θ are shown in Figure 1. Comparing the XRD peak information of MCP and ICP, the related peaks were well matched to those of pyrolusite (δ-MnO_2_) and goethite (α-FeOOH), respectively [22]. The SEM photographs of the acid-washed natural pumice and MCP are also shown in Figures 2a and 2b, respectively. Figure 2a shows micropores on natural pumice surface which are filled and closed with manganese oxides in MCP as presented in Figure 2b. The SEM results of the origin pumice and iron coated pumice by Kitis *et al.*[15] were almost similar to those of the present study. The BET surface area of ICP and MCP measured were 3 and 1 m^2^/g, respectively.

### Batch experiments

**Figure 3:**
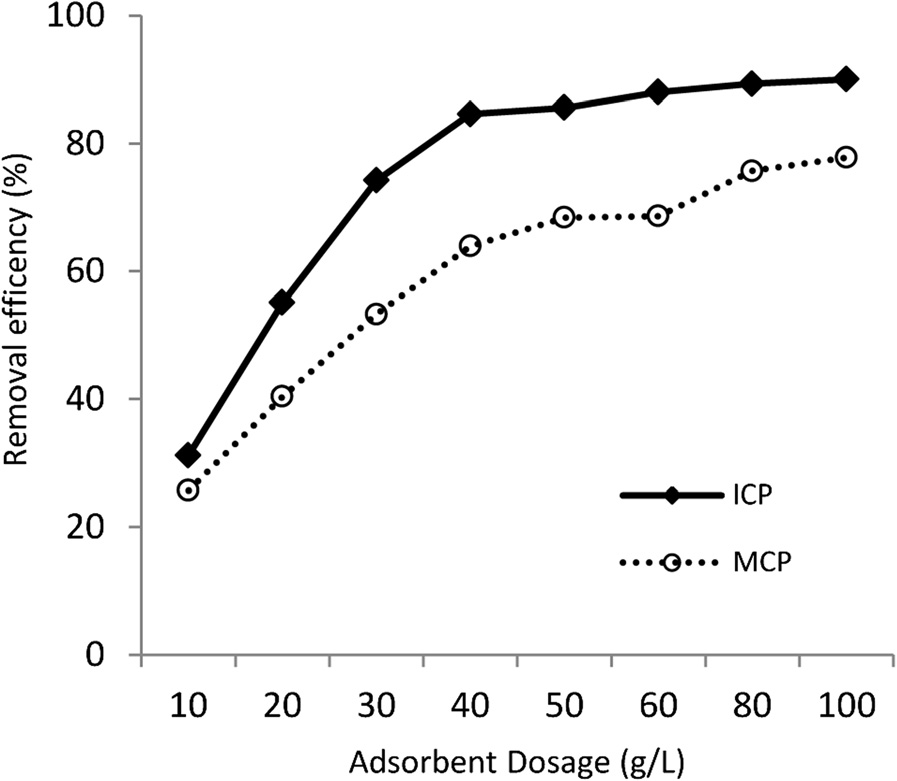
The effect of the adsorbents dose on As(V) removal, temperature: 22±1°C, As(V) initial concentration: 1000 μg/L, pH=7, contact time: 2hrs.

**Figure 4:**
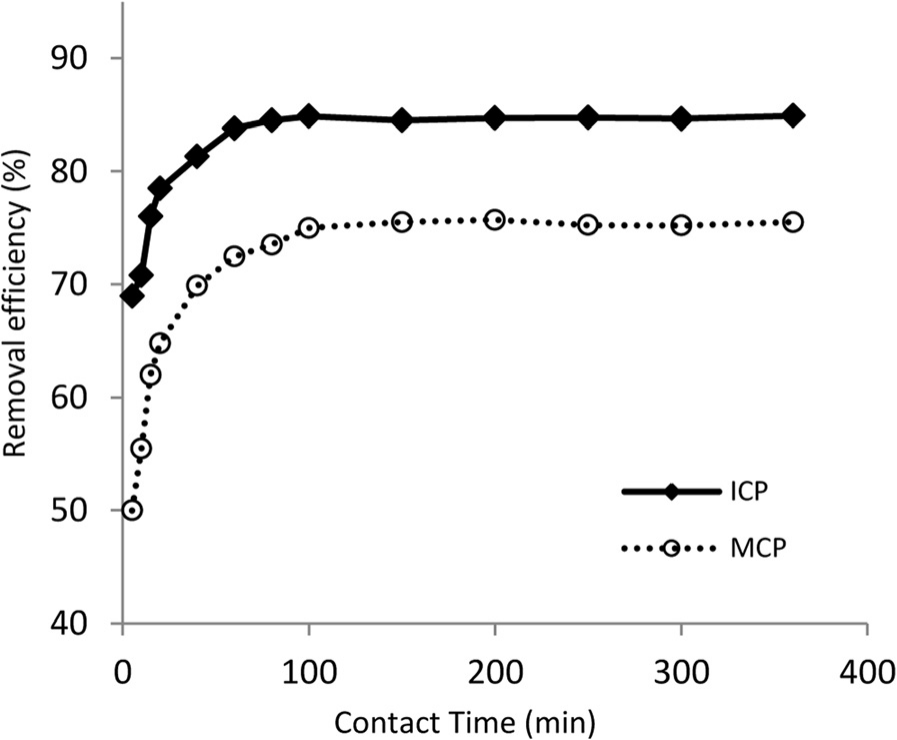
The effect of contact time on As(V) removal, temperature: 22±1°C, initial As(V) concentration: 1000 μg/L, pH=7, adsorbents dose: 40 g/L for ICP and 80 g/L for MCP.

**Figure 5:**
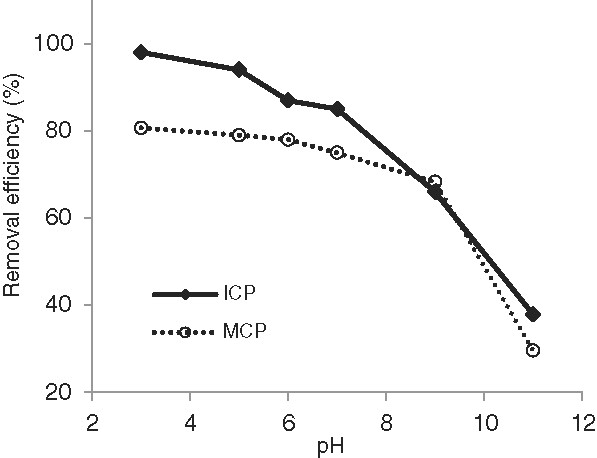
The effect of pH on As(V) removal, temperature: 22±1°C, initial As(V) concentration: 1000 μg/L, contact time: 80 min for ICP and 100 min for MCP, adsorbents dose: 40 g/L for ICP and 80 g/L for MCP.

**Figure 6:**
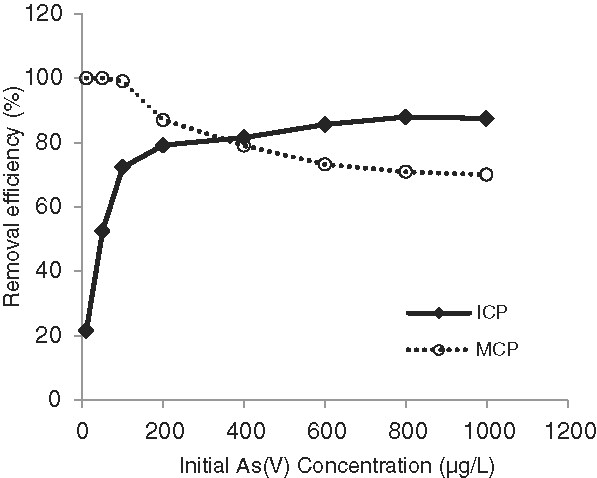
The effect of initial As(V) concentration on arsenate removal, temperature: 22±1°C, pH=7, contact time: 80 min for ICP and 100 min for MCP, adsorbents dose: 40 g/L for ICP and 80 g/L for MCP.

Figure 3 illustrates the effect of varying dosages of ICP and MCP at a range of 10 to 100 g/L on arsenate removal from solution containing 1000 μg/L As(V) at 2hrs contact time under pH=7. The removal efficiency of 1000 μg/L As(V) using 40 g/L ICP and 80 g/L MCP were investigated by varying the contact time from 5 to 360 min at pH 7 and it is shown in Figure 4. In order to elucidate the mechanism of As(V) adsorption by ICP and MCP, arsenate removal by the solids were evaluated in a wide range of pH from acidic (pH=3) to alkaline (pH=11), which is exhibited in Figure 5. The initial concentration of As(V) is another important parameter which affects the adsorption process. The As(V) adsorption on ICP and MCP as a function of initial concentration of As(V) is shown in Figure 6.

### Sorption kinetic

**Figure 7:**
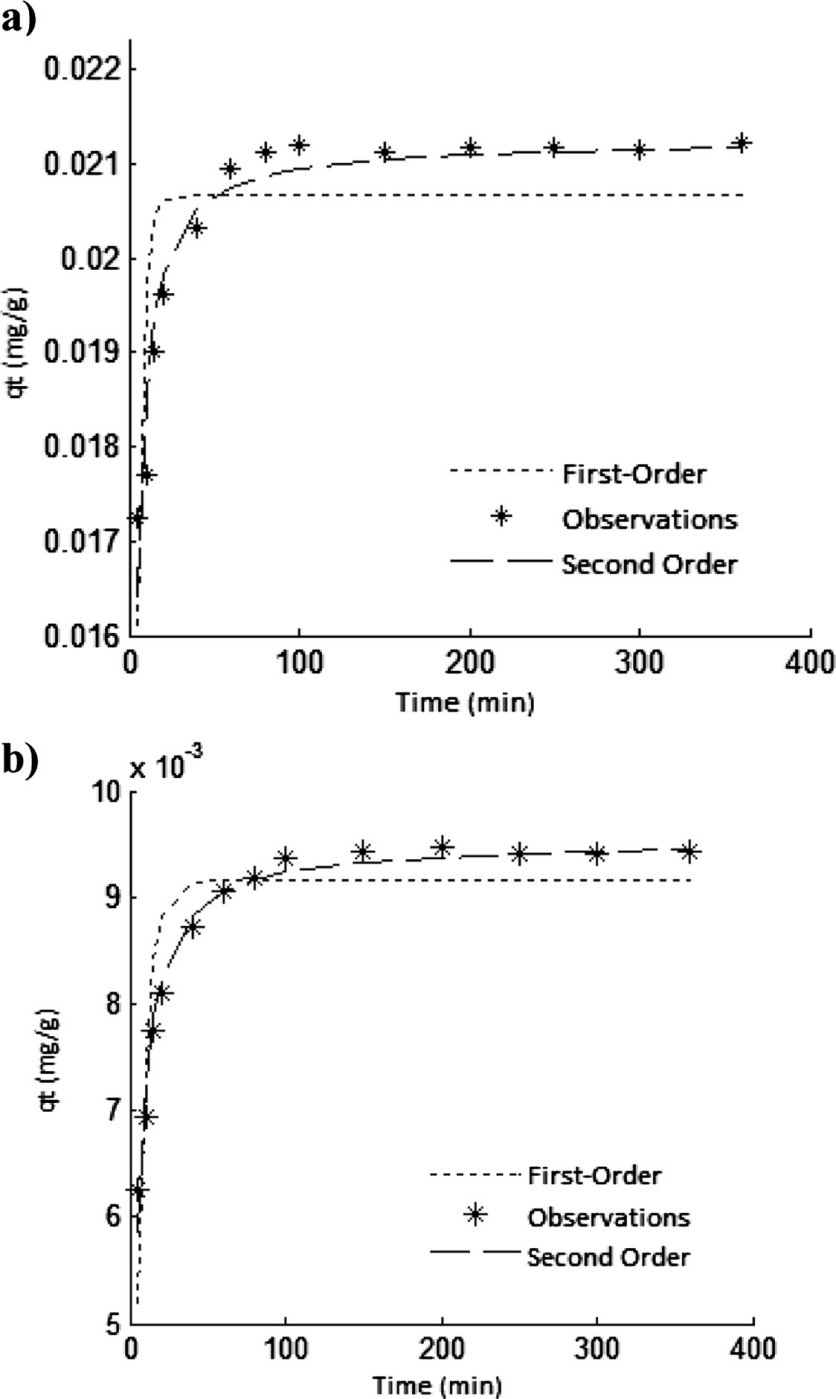
Kinetic plots for As(V) adsorption on ICP (a) and MCP (b), temperature: 22±1°C, initial As(V) concentration: 1000 μg/L, pH=7, (a) adsorbents dose: 40 g/L, (b) adsorbents dose: 80 g/L.

Figure 7a and b show that the pseudo-first order and pseudo-second order kinetic models fit for As(V) adsorption on ICP and MCP, respectively. The first order and second order rate constants accompanied by RMSE values are represented in Table 1.

**Table 1:** Parameters of kinetic models for As(V) adsorption onto ICP and MCP using non-linear method

**Kinetic models**	**Model parameters**	**Adsorbents**	
		**ICP**	**MCP**
	k_1_ (1/min)	0.021	0.009
Pseudo-fist-order	q_e_ (mg/g)	0.302	0.167
	RMSE	0.00096	0.00052
	k_2_ (g/mg.min)	33.591	32.370
pseudo-second-order	q_e_ (mg/g)	0.021	0.010
	RMSE	0.00039	0.00018

### Sorption isotherm

**Figure 8:**
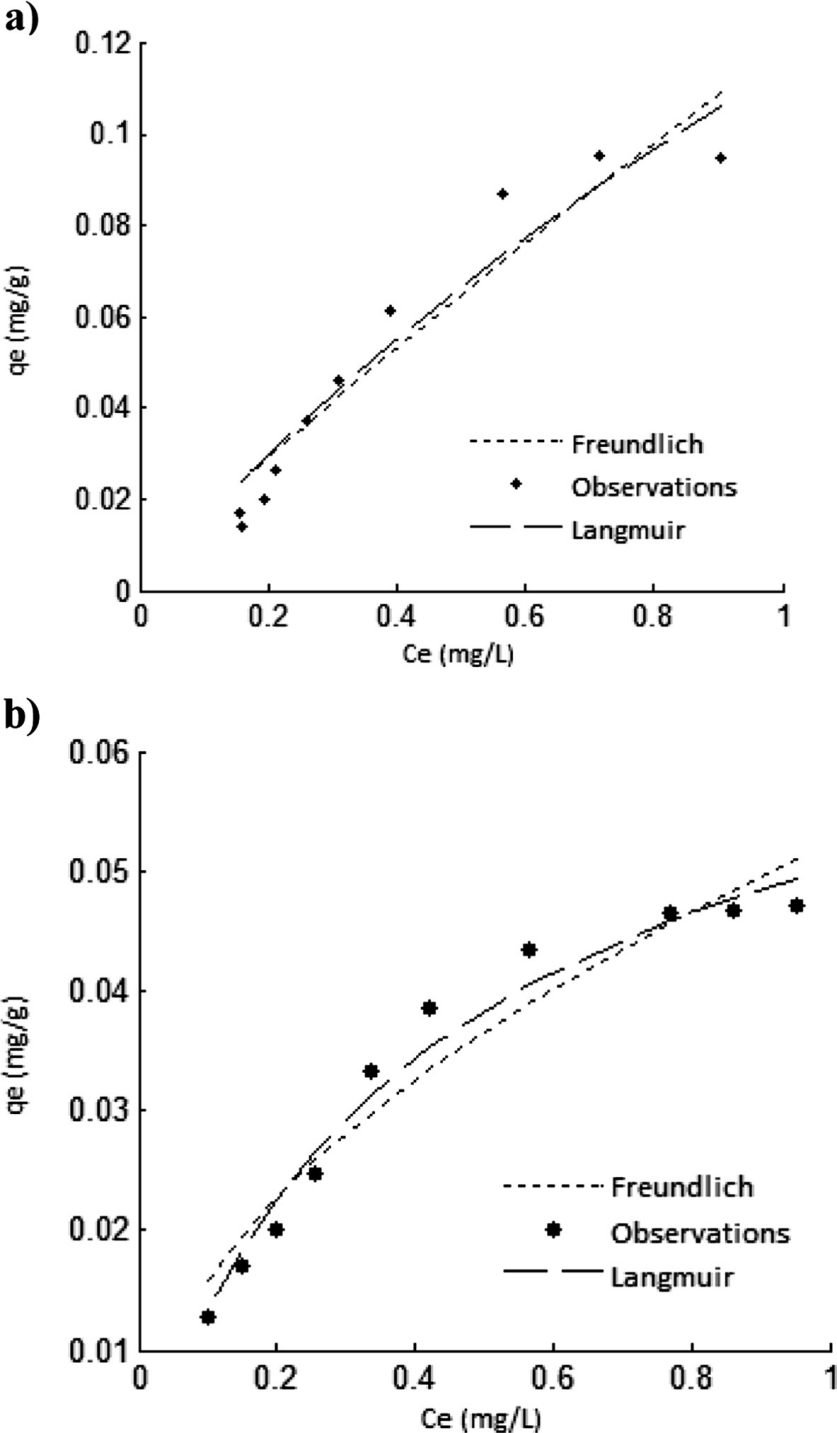
Isotherm plots for As(V) adsorption on ICP (a) and MCP (b), temperature: 22±1°C, initial As(V) concentration: 1000 μg/L, pH=7, contact time: 24hrs.

The isotherm plots of adsorption As(V) onto ICP and MCP are given in Figure 8a and b, respectively. The non-linear parameters of these models are listed in Table 2.

## Discussion

### The effect of adsorbent dose

Figure 3 shows that the removal efficiency of As(V) significantly rose to 84.6% level with every increase in the ICP dose from 10 to 40 g/L. Thereafter, with the ICP amounts more than 40 g/L the removal efficiency did not show significant increase. An almost similar trend was also obtained for As(V) removal by MCP although the removal efficiency only rose to 77.8% as solid dose increased from 10 to 100 g/L. As mentioned above, with increasing of amounts of adsorbents, the number of active adsorptive sites available for As(V) ions also rose showing an increase in the uptake of As(V) at first (Bulut and Aydin, [23]; Wan Ngah and Hanafiah, [24]). However, it was observed that the As(V) uptake did not rise following further increase of the solids dose because of the low residual As(V) concentration in solution. According to the results, the dosage of ICP and MCP that were chosen for the experiments were 40 g/L and 80 g/L, respectively.

### The effect of contact time

Figure 4 exhibits that the As(V) adsorption by adsorbents process takes place in two main phases. The first phase was related to the rapid As(V) uptake by both adsorbents within 20 min contact time while the second phase followed a slower adsorption rate which achieved to the equilibrium latter. The rapid uptake by adsorbents in the first phase is mainly because of the unsaturated adsorptive sites which have been rapidly occupied by As(V) anions at the beginning of the process [7]. The equilibrium time for As(V) adsorption on the adsorbents obtained after 80 and 100 min for ICP and MCP, respectively.

### The effect of pH

As Figure 5 shows As(V) adsorption by both adsorbents were pH dependent. Whereas As(V) adsorption on both solids was maximum in acidic pH and gradually decreased with increasing pH to neutral and then alkaline values. The As(V) removal efficiency by ICP and MCP obtained at pH=3 was up to 98% and 87% while the efficiency descended to 83.3% and 76.1%, respectively at pH=7 which is in pH range of drinking waters. The diversity in As(V) adsorption on the solids’ surface at different pH values are attributed to the surface charge of adsorbent and As(V) speciation (Tuutijarvi *et al.*, [4,25]).

The pH_ZPC_ values of pure iron oxides are between 7.4 and 8.7 [26]. Such range was found to be between 6.9 and 9.3 for uncoated pumice and 5 to 8.4 for iron coated pumice [15]. In addition, the amounts of pH_ZPC_ of activated alumina and manganese oxide-coated alumina were found to be 8.25 and 7.5, respectively [12]. Thus, it can be concluded that the coating material covers the surface electrical properties of the support media [15].

In aqueous systems, hydrolysis of Fe(III) products (FeOH^+2^, Fe(OH)_2_^+^, Fe(OH)_3_^0^, Fe(OH)_4_^-^) depend on the solution pH (Katsoyiannis and Zouboulis, [27]). Thus, over these pH_ZPC_ values, iron hydroxides are present in the monomeric anionic form of Fe(OH)_4_^-^, hence they repulse the arsenate anions (HAsO_4_^-2^ and AsO_4_^-3^). Consequently, As(V) adsorption by ICP reduces in alkaline pH. As mentioned above, the removal of As(V) by ICP was very high in acidic pH. Since the predominant species of As(V) in pH=2.3-6.9 is H_2_AsO_4_^-^[7], it can be effectively removed by the iron hydroxides, which in this pH range are present as cationic monomers of FeOH^+2^ and Fe(OH)_2_^+^. When the solution pH increases from low pH to pH_ZPC_, the reduction of As(V) adsorption by MCP is attributed to the decreasing electrostatic attraction between the surface of solid and anionic arsenate species. The lower adsorption of As(V) at pH values more than pH_zpc_ is because of an increased repulsion between the anionic As(V) species and negatively charged sites on the surface of MCP (Liu and Zhang, [28,29]). The responsible mechanism for As(V) removal by ICP and MCP was adsorption on solids, which is refered to the formation of surface complexes between soluble As(V) species and the solid hydroxide surface sites. The sorption process of As(V) by ICP is indicated schematically as equation (6) (Katsoyiannis and Zouboulis, [27]):

(6) $$P–\mathrm{FeOH}+H_{2}{{\mathrm{AsO}}_{4}}^{-}\to P–\mathrm{Fe}–{{\mathrm{HAsO}}_{4}}^{-}+H_{2}O$$

Also, the possible reaction between As(V) and MCP is shown as equation (7):

(7) $$\begin{array}{l} P-\mathrm{Mn}{\left(\mathrm{OH}\right)}_{2}+H_{2}\mathrm{As}{O_{4}}^{-}\to P-\mathrm{Mn}\left(\mathrm{OH}\right)\\ \;-\mathrm{HAs}{O_{4}}^{-}+H_{2}O \end{array}$$

### The effect of initial As(V) concentration

Figure 6 shows when the initial As(V) concentration in synthetic water was slight, namely 10 and 50 μg/L, ICP efficiency was 21.5% and 52.5%, respectively. But ICP efficiency noticeably increased following a little increase in the initial concentration of As(V). This increase might be due to the high possibility of collision between arsenic ions and the surface of adsorbent (Wan Ngah and Hanafiah, [24]). Subsequently, the arsenate uptake did not change significantly for increases in the initial concentration of As(V) more than 600 μg/L. It could be for this reason that surface of adsorbent was saturated by As(V) anions. In contrast, MCP almost completely removed the low initial concentrations of As(V) (10, 50 and 100 μg/L), because there are more sites with the MCP for As(V) anions adsorption [16]. However, the performance of MCP gradually declined with increasing concentration of As(V).

### Sorption kinetics

It is apparent from Table 2 that both models acceptably have predicted the data. However, relatively lower RMSE was obtained for the pseudo-second order kinetic model. From comparing K_2_ values obtained for the solids, it is obvious that adsorption rate of As(V) onto the solids is almost same but it was slightly rapid on ICP. Also, q_e_ constant for ICP was more than that of MCP. It means that, sorption capacity of ICP for As(V) adsorption is more than that of MCP.

**Table 2:** Isotherm constants for As(V) adsorption on ICP and MCP by non-linear method

**Isotherm models**	**Model parameters**	**Adsorbents**	
		**ICP**	**MCP**
	q_m_ (mg/g)	0.387	0.072
Langmuir	b (L/mg)	2.280	0.415
	RMSE	0.0089	0.0022
	k_f_ (mg/g) (mg/L)^-1/n^	0.119	0.052
Freundlich	n	1.142	1.930
	RMSE	0.0098	0.0036

### Sorption isotherm

It is evident from the RMSE value that for both ICP and MCP, the experimental data fitted well to Langmuir model (Table 1) as b value obtained for ICP was more than for MCP which means As(V) adsorption bond with ICP is stronger than with MCP. The maximum adsorption capacity (q_m_) for ICP was 1.01 and for MCP was 0.07. It shows that ICP have more adsorption capacity than MCP, so it is a better adsorbent for As(V). This might be because that the affinity of Fe with As(V) anion is more and the second reason can be that ICP has the more specific surface area than MCP. Based on the assumption of the Langmuir isotherm, it can be estimated that both ICP and MCP should have mainly homogeneous sites.

Comparing to some adsorbents such as Mn-oxide coated alumina, maghemite nanoparticles, iron and aluminium oxides ( [12,21]; Tuutijarvi, *et al.*, [4]) which have high As(V) adsorption capacity, the adsorption capacities of ICP and MCP are low. However, compared to some other adsorbents that have lower As(V) adsorption capacities such as Fe-oxide loaded sand, Mn-oxide loaded sand [11], ICP can remove As(V) from water more efficiently.

## Conclusion

According the results of this study, the iron-coated pumice (ICP) and manganese-coated pumice (MCP) were found to be efficient and inexpensive adsorbents for As(V) removal from aqueous solutions whereas ICP and MCP were able to remove 98% and 87% of As(V), respectively, at an initial concentration of 1000 μg/L in pH=3 within a short contact time. In addition, the noticeable uptake was observed by both adsorbents at pH=7, as well. Therefore, depending on the contamination rate, it is recommended to apply ICP and MCP for high and low contamination rates of As(V) in aqueous solutions, respectively.

## Competing interests

The authors declare that they have no competing interests.

## Authors’ contributions

LBF performed all the experiments and drafted the manuscript. BS supervised all the experiments and also read and edited the manuscript. MH advised the experimental methods applied and confirmed accuracy of the results obtained. RKh was involved in discussion of the results. All authors read and approved the final manuscript.
